# A TBX2-HLX Regulatory Axis Is Associated with Advanced Prostate Cancer

**DOI:** 10.3390/biomedicines14081865

**Published:** 2026-08-20

**Authors:** Murugananthkumar Raju, Philip Irwin Motakatla, Hamed Khedmatgozar, Raaghav Nandana, Dongming Jiang, Zheyun Niu, Rozina Vafa, Sayanika Dutta, Manisha Tripathi

**Affiliations:** 1 Department of Cell Biology and Biochemistry, Texas Tech University Health Sciences Center, Lubbock, TX 79430, USA; 2 Lubbock High School, Lubbock, TX 79401, USA; 3 Department of Urology, Texas Tech University Health Sciences Center, Lubbock, TX 79430, USA

**Keywords:** castration-resistant prostate cancer, homeobox transcription factor, NOTCH signaling, extracellular matrix, metastasis, biomarker, epithelial–mesenchymal transition, transcriptional regulation

## Abstract

**Background:** Homeobox transcription factors regulate developmental programs, cellular plasticity, and tumor progression, yet the role of H2.0-like homeobox (HLX) in prostate cancer (PCa) remains poorly defined. We investigated the clinical significance of HLX and its relationship to the pro-metastatic transcription factor TBX2. **Methods:** Transcriptomic and clinical datasets from TCGA, MET500, and SU2C/PCF cohorts were analyzed to assess HLX expression, clinicopathologic associations, and its relationship with TBX2. Functional studies in human PCa cell lines included TBX2 gain- and loss-of-function, HLX knockdown, chromatin immunoprecipitation (ChIP), and expression analyses. Shared HLX- and TBX2-associated pathways were evaluated by Reactome enrichment analysis, and Hallmark Gene Set Enrichment Analysis compared castration-resistant prostate cancer (CRPC) bone metastases with high versus low HLX expression (GSE77930; *n* = 5/group). *In vivo* relevance was assessed in an orthotopic TBX2 dominant-negative PCa xenograft model. **Results:** Human PCa datasets showed that HLX expression was elevated in PCa versus normal prostate tissue and associated with higher Gleason grade, lymph node involvement, aggressive molecular subtypes, and shorter disease-free survival. HLX expression also positively correlated with TBX2 across human PCa cohorts. HLX- and TBX2-associated transcriptional programs converged on extracellular matrix organization, cell adhesion, NOTCH, and VEGF-MAPK signaling pathways. Furthermore, HLX-high CRPC bone metastases were enriched for epithelial–mesenchymal transition, NOTCH, TGF-β, inflammatory, angiogenic, hypoxic, and KRAS signaling pathways. Mechanistic studies showed that HLX knockdown suppressed extracellular matrix-associated genes and key NOTCH pathway components. ChIP demonstrated direct TBX2 binding to the HLX promoter, and genetic modulation of TBX2 expression established HLX as a downstream target of TBX2. Consistent with these findings, reduced HLX expression in orthotopic TBX2 dominant-negative xenografts was associated with loss of metastatic progression. **Conclusions:** HLX is a candidate biomarker of aggressive PCa and a direct transcriptional target of TBX2. These findings identify a previously unrecognized TBX2–HLX regulatory axis associated with metastatic transcriptional programs and aggressive disease in advanced PCa.

## 1. Introduction

Prostate cancer (PCa) is the most frequently diagnosed malignancy among men in the United States (excluding skin cancers) and remains a leading cause of cancer-related mortality worldwide [1]. Although localized disease can often be effectively managed with current therapeutic approaches, progression to advanced disease—defined as metastatic castration-resistant prostate cancer (CRPC)—remains a major clinical challenge and is associated with poor survival outcomes [2,3]. Advanced PCa is characterized by extensive transcriptional reprogramming that enables tumor cells to adapt to therapeutic pressure and hostile microenvironments [4,5]. Gene expression profiling studies of advanced PCa have revealed widespread alterations in pathways governing cellular growth, survival, migration and lineage plasticity [6,7,8,9]. These transcriptional changes contribute to metastatic progression and therapeutic resistance, underscoring the need to identify the key transcriptional regulators that drive aggressive disease states and may represent therapeutic vulnerabilities [4,7,10].

Homeobox transcription factors are developmental regulators that control cell growth, differentiation, and tissue identity during embryogenesis. Dysregulated expression of these factors has been implicated in cancer progression through the promotion of proliferation, impaired differentiation, cellular plasticity and metastatic behavior [11,12,13]. The H2.0-like homeobox transcription factor HLX, a member of the NK-like (NKL) subclass of homeobox genes, was initially characterized for its role in immune development and lineage specification [14,15,16]. More recently, HLX has emerged as a context-dependent regulator of tumor progression. In acute myeloid leukemia (AML), HLX is overexpressed and functions as an oncogenic driver that promotes proliferation, blocks differentiation, and correlates with adverse clinical outcomes [15,17]. Aberrant HLX expression has also been reported in Hodgkin lymphoma and other hematologic malignancies [18,19,20]. In solid tumors, HLX has been linked to advanced disease, cellular plasticity, and poor prognosis and has been implicated in developmental programs that regulate cell migration, angiogenesis, and vascular remodeling [21]. Collectively, these observations suggest that HLX regulates transcriptional programs linked to lineage identity and cellular plasticity, processes that are increasingly recognized as drivers of aggressive cancer phenotypes. Consistent with the broader importance of developmental transcriptional regulators in prostate tumorigenesis, germline mutations in the homeobox transcription factor HOXB13, particularly the G84E variant, are strongly associated with hereditary and early-onset PCa risk [22,23]. These findings underscore the importance of developmental transcription factor networks in PCa biology and provide a rationale for investigating the role of HLX in disease progression.

Further supporting an oncogenic role for HLX, recent studies in group 3 medulloblastoma identified an lnc-HLX-2-7/HLX signaling axis that drives tumor progression through activation of MYC-dependent transcriptional programs [24]. Among these targets, the T-box transcription factor TBX2, is of particular interest in PCa. TBX2 is a developmental transcription factor that functions as a transcriptional and epigenetic regulator and has been implicated in oncogenesis across multiple malignancies [25,26]. Our previous studies demonstrated that TBX2 is a potent driver of PCa progression, promoting metastatic dissemination, lineage plasticity, and therapeutic resistance [27,28,29]. Mechanistically, TBX2 promotes aggressive tumor behavior by suppressing tumor suppressor pathways, inducing epithelial–mesenchymal transition, and activating oncogenic transcriptional programs associated with invasion and metastasis [30,31,32]. Beyond PCa, TBX2 contributes to tumor progression in breast, pancreatic, melanoma, and other epithelial cancers, where it promotes cellular plasticity, invasion, and resistance to senescence [26,33,34,35]. Despite its established role as a master regulator of aggressive disease, the downstream effectors through which TBX2 mediates these functions remain incompletely defined.

Building on extensive efforts from our laboratory to better understand the role of TBX2 in PCa, which have established it as a master regulator of metastatic progression, lineage plasticity, and therapeutic resistance, we sought to identify novel downstream transcriptional targets that may contribute to aggressive disease-associated programs. In this study, we investigate the clinical significance of HLX in PCa, characterize its relationship with TBX2 across multiple patient cohorts and experimental models, and identify HLX as a transcriptional target of TBX2. Collectively, our findings define a previously unrecognized TBX2-HLX regulatory axis associated with aggressive and metastatic PCa progression.

## 2. Materials and Methods

### 2.1. Clinical and Transcriptomic Data Acquisition

Transcriptomic and associated clinical data for normal prostate tissue and prostate adenocarcinoma (PRAD) were obtained from The Cancer Genome Atlas (TCGA) through the UALCAN database (https://ualcan.path.uab.edu/, accessed on 1 November 2025) [36,37]. All gene expression analyses were performed using the normalized expression values and default analytical parameters provided by the respective databases unless otherwise specified. No additional normalization was performed. Normalized HLX gene expression data, molecular subtype annotations, Gleason scores, TP53 mutation status, and nodal metastasis status were retrieved using the UALCAN platform. In the MET500 metastatic PCa cohort, HLX expression levels were compared between ERG fusion-positive and ERG fusion-negative tumors. All expression and clinical association plots were generated using the UALCAN web-based analysis tools. Disease-free survival analyses were performed using the GEPIA web server based on TCGA-PRAD data. Patients were stratified into HLX-high and HLX-low groups using the median expression cut-off (50%:50%). Survival curves were generated using the Kaplan–Meier method, and statistical significance was determined by the log-rank test. Hazard ratios were estimated using the Cox proportional hazards (Cox PH) model implemented in GEPIA (http://gepia.cancer-pku.cn/, accessed on 1 November 2025) [38].

### 2.2. cBioPortal Database Analysis

cBioPortal (The cBio Cancer Genomics Portal; http://www.cbioportal.org/, accessed on 1 November 2025) is an open-access, web-based platform used to explore and analyze large-scale cancer genomics datasets [39,40]. The following human clinical datasets were accessed for correlation analyses between HLX and TBX2 mRNA gene expression: PRAD (TCGA, Firehose Legacy; *n* = 491, [41]), PRAD (Fred Hutchinson Cancer Research Center (CRC), Nature Medicine 2016; *n* = 133, [42]), Metastatic PRAD (SU2C/PCF Dream Team, PNAS 2019; *n* = 208, [43]), and Metastatic PCa (SU2C/PCF Dream Team, Cell 2015; *n* = 118, [44]). Pairwise correlations between HLX and TBX2 expressions across TCGA, Fred Hutchinson, and SU2C/PCF datasets were assessed using Spearman correlation analysis within the cBioPortal platform [39,40]. The gene name “HLX” was used to query correlated expression with “TBX2”, and scatter plots were generated directly using the cBioPortal. The cBioPortal database was accessed between November 2025 and February 2026.

### 2.3. Co-Expression and Reactome Pathway Enrichment Analyses of Human PCa Datasets

Gene co-expression analyses were performed using the cBioPortal platform on metastatic PRAD samples from the SU2C/PCF Dream Team dataset (PNAS, 2019) [39,40,43]. For each gene of interest (HLX and TBX2), genes exhibiting positive co-expression were identified using Spearman correlation analysis. Genes meeting the thresholds of Spearman correlation coefficient ≥ 0.4 and a false discovery rate-adjusted *p*-value < 0.05 were retained for downstream analyses. Reactome pathway enrichment analysis was performed independently for HLX- and TBX2-correlated gene sets using the Reactome pathway database. Enriched pathways were filtered based on statistical significance (adjusted *p*-value < 0.05). Pathway enrichment results were visualized as dot plots using the ggplot2 package (version 4.0.3) in RStudio (version 4.1) for data visualization (https://cran.r-project.org/web/packages/ggplot2/index.html, accessed on 15 December 2025). Overlap between HLX- and TBX2-associated pathways was assessed, and shared pathways were visualized using Venn diagrams to identify convergent biological programs.

### 2.4. Cell Lines and Growth Conditions

Human PCa cell lines LNCaP, PC3, C4-2B, and DU145 were obtained from the American Type Culture Collection (ATCC). 22Rv1 and VCaP cell lines were obtained from the laboratory of Leland W. K. Chung, Uro-Oncology research program, Department of Medicine, Cedars-Sinai Medical Center, Los Angeles, CA. LNCaP enzalutamide-resistant cells (LNCaP^EnzaR^) were kindly provided by Robert J. Matusik and Renjie Jin, Vanderbilt University Medical Center (VUMC), Nashville, TN. C4-2B enzalutamide-resistant cells (C4-2B^EnzaR^) were kindly provided by Allen Gao, Department of Urologic Surgery, University of California Davis, Sacramento, CA. C4-2B^EnzaR^, LNCaP, LNCaP^EnzaR^, and the 22Rv1 cells were maintained in Roswell Park Memorial Institute medium 1640 (RPMI-1640; Corning, Corning, NY, USA, Cat #10-040-CV) supplemented with 10% fetal bovine serum (FBS; Gibco, Waltham, MA, USA, Cat #16000044) and 1% penicillin–streptomycin (PS; Cytiva, Wilmington, DE, USA, Cat #SV30010). LNCaP^EnzaR^ and C4-2B^EnzaR^ cells were continuously cultured in the presence of 10 µM enzalutamide (Selleckchem, Houston, TX, USA, Cat #MDV3100). PC3, C4-2B, and VCaP cells were cultured in Dulbecco’s Modified Eagle Medium (DMEM; Corning, Corning, NY, USA, Cat #10-013-CV) supplemented with 10% FBS and 1% PS. DU145 cells were grown in Corning™ Minimum Essential Medium Eagle 1× (MEM) with Glutamine (Corning, Corning, NY, USA, Cat #10-009-CV) supplemented with 10% FBS and 1% PS. All cell lines were maintained at 37 °C in a humidified incubator with 5% CO_2_ and routinely tested for mycoplasma contamination.

### 2.5. Plasmid Isolation, Transduction and Genetic Modulation of TBX2 and HLX in Human PCa Cells

Plasmids were isolated using the QIAfilter Plasmid Midi Kit (Qiagen, Cat #12243). Plasmid quality and concentration were assessed using a NanoDrop™ One/One^C^ Microvolume UV-Vis Spectrophotometer (Thermo Fisher Scientific, Waltham, MA, USA). PC3, C4-2B, and DU145 cells were transduced with either pBABE-puro-Neo or pBABE-puro-TBX2-DN constructs, generously provided by Colin Goding (Nuffield Department of Medicine, University of Oxford, UK). LNCaP cells were stably transduced with either a control vector (pBS-EF1-IRES-NEO-Control; Addgene, Watertown, MA, USA, Cat #80757) or a TBX2 overexpression vector (LZRS-TBX2-IRES-GFP), generously provided by Maarten van Lohuizen (Netherlands Cancer Institute, Amsterdam, The Netherlands). PC3 cells were stably transduced with an shHLX plasmid (Genecopeia, Rockville, MD, USA, Cat # HSH100211-LVRU6GP) or corresponding control plasmid (Cat # CSHCTR001-LVRU6GP). Retroviral particles were generated by Lipofectamine 2000–mediated transfection of 0.8 × 10^6^ 293FT cells in a 60 mm dish with the indicated retroviral constructs together with the pCL-Ampho retroviral packaging vector (Novus Biologicals, Centennial, CO, USA, Cat. #NBP2-29541). Viral supernatants were collected 36 h post-transfection, centrifuged at 300× *g* for 5 min, filtered through a 0.45 µm membrane, mixed 1:1 with fresh culture medium containing 10 µg/mL polybrene (Sigma-Aldrich, St. Louis, MO, USA, Cat. #TR-1003-G) and then used to infect target cells. Target cells were infected in three consecutive rounds, followed by puromycin selection (5 µg/mL for PC3 and 2 µg/mL for C4-2B and DU145 cells). GFP-positive LNCaP^TBX2OE^ cells were isolated by flow cytometry. TBX2 dominant-negative mediated activity disruption and TBX2 overexpression were validated by Western blot analysis, while HLX knockdown in PC3 cells was confirmed by qPCR analysis.

### 2.6. Cell Lysate Preparation and Western Blotting

Cells were harvested and lysed in RIPA buffer (ThermoFisher Scientific, Waltham, MA, USA, Cat #89901) containing 1× protease and phosphatase inhibitors (PPI, Halt™ Protease and Phosphatase Inhibitor Cocktail 100×, Cat #78442). Protein concentration was determined using the DC™ Protein Assay Kit I (Bio-Rad, Hercules, CA, USA, Cat #5000111). Equal protein amounts (60 µg) were loaded onto SDS-PAGE gels (Bio-Rad, Cat #4561084), followed by protein transfer to PVDF membranes (Millipore Sigma, Burlington, MA, USA, Cat #IPVH00010). Membranes were blocked with 1× Blocker™ FL Fluorescent Blocking Buffer (Thermo Fisher Scientific, Waltham, MA, USA, Cat #37565) for 1 h. After blocking, membranes were incubated overnight at 4 °C with primary antibodies specific to the target proteins. Following three washes with 1× TBS-T (15 min each), membranes were incubated with HRP-conjugated secondary antibodies for 1 h at room temperature (RT) and then washed three times with 1× TBS-T (15 min each). Protein bands were visualized using SuperSignal™ West Pico PLUS Chemiluminescent Substrate (Thermo Fisher Scientific, Waltham, MA, USA, Cat #34580), and densitometry analysis was conducted with ImageJ software version 1.54g (NIH). Antibodies used included: HLX (Proteintech, Rosemont, IL, USA, Cat #14336-1-AP), TBX2 (Santa Cruz Biotechnology (SCBT), Dallas, TX, USA, Cat #sc-514291), Anti-β-actin (SCBT, Cat #sc-47778). HRP-conjugated secondary antibodies were purchased from Cell Signaling Technology, Danvers, MA, USA (anti-rabbit, Cat #7074; anti-mouse, Cat #7076).

### 2.7. cDNA Synthesis and qPCR Analysis

RNA was isolated from human PCa cell lines using the RNeasy Kit (QIAGEN, Hilden, Germany, Cat #74106). cDNA was synthesized from 2 µg of total RNA using the High-Capacity RNA-to-cDNA Kit (Applied Biosystems, Waltham, MA, USA, Cat #4368813). qPCR was performed in biological triplicates using PowerUp SYBR Green Master Mix (Thermo Fisher Scientific, Waltham, MA, USA, Cat #A25742). Gene-specific primers for HLX and TBX2 (listed in Supplementary Table S2) were synthesized by Integrated DNA Technologies (IDT, Coralville, IA, USA). qPCR reactions were run on a QuantStudio 12K Flex Real-Time PCR System. Relative mRNA expression levels were normalized to β-actin and calculated using the 2^−ΔCt^ method.

### 2.8. Chromatin Immunoprecipitation (ChIP) Assay

Putative TBX2-binding sites within the HLX promoter were identified using JASPAR (https://jaspar.elixir.no/, accessed on 1 November 2025) [45] and the Eukaryotic Promoter Database (https://epd.expasy.org/epd/, accessed on 2 November 2025) [46,47]. ChIP assays were performed as previously described [29], with minor modifications. PC3 cells were grown in 150 mm culture dishes to approximately 80% confluency and crosslinked with formaldehyde at a final concentration of 1% for 8 min at RT with gentle shaking, followed by quenching with 0.125 M glycine for 5 min at RT. Cells were then washed three times with ice-cold 1× PBS, collected in PBS supplemented with 1× PPI, and pelleted by centrifugation at 300× *g* for 5 min. Cell pellets were lysed in ChIP lysis buffer (50 mM Tris-HCl, pH 8.0; 10 mM EDTA; 1% SDS) containing 1× PPI. Chromatin was fragmented by sonication using a Bioruptor (Diagenode Inc., Denville, NJ, USA) for 24 cycles (30 s on/30 s off). Soluble chromatin was quantified, and 100 µg was incubated with 1 µg of TBX2 antibody (SCBT) or corresponding mouse IgG control (Cell Signaling Technology, Danvers, MA, USA) overnight at 4 °C on a nutating mixer. The next day, chromatin-antibody complexes were captured by incubation with Dynabeads Protein A/G (Invitrogen, Carlsbad, CA, USA, Cat #10003D) for 2 h at 4 °C. Immunoprecipitates were washed sequentially with low-salt wash buffer (20 mM Tris-HCl, pH 8.1; 0.1% SDS; 1% Triton X-100; 2 mM EDTA; 150 mM NaCl), high-salt wash buffer (identical composition with 500 mM NaCl), and TE buffer (10 mM Tris-HCl, pH 8.0; 1 mM EDTA). Crosslinks were reversed by overnight incubation at 65 °C in the presence of 5 M NaCl, followed by sequential treatment with RNase A (Millipore Sigma, Burlington, MA, USA, Cat# 70856) and proteinase K (Thermo Fisher Scientific Waltham, MA, USA, Cat# EO0491). ChIP-enriched DNA was purified using a PCR purification kit (Qiagen, Hilden, Germany, Cat# 28104) and analyzed by both PCR and qPCR (*n* = 3) using specific primers listed in Supplementary Table S2.

### 2.9. Hallmark Gene Set Enrichment Analysis Comparing HLX-High and HLX-Low Human CRPC Bone Metastases

Transcriptomic data from the GSE77930 dataset was retrieved from the Gene Expression Omnibus (GEO) database and included 19 CRPC bone metastasis samples. Samples were stratified according to HLX expression levels using quartile-based grouping. Samples within the upper quartile of HLX expression were defined as the HLX-high group (*n* = 5), whereas samples within the lower quartile were defined as the HLX-low group (*n* = 5). Differential gene expression analysis was performed between HLX-high and HLX-low groups, and genes were ranked based on their differential expression profiles. Gene Set Enrichment Analysis (GSEA) was subsequently performed using the Molecular Signatures Database (MSigDB) Hallmark gene sets. Pathways with an adjusted *p*-value < 0.05 were considered significantly enriched. All statistical analyses and visualizations were performed using R software (version 4.6.1).

### 2.10. Immunohistochemistry (IHC)

All animal studies were conducted in accordance with protocols approved by the Institutional Animal Care and Use Committee (IACUC: 18038). Luciferase-tagged PCa cells (PC3^Neo^ or PC3^TBX2DN^) were orthotopically engrafted (1 × 10^6^ cells per mouse) into the anterior prostate lobes of 4–6-week-old athymic nude mice, and xenograft tumors were harvested after 10 weeks [27]. To assess HLX expression in PC3^TBX2DN^ and PC3^Neo^ xenograft tissues, slides were stained using the anti-HLX antibody. Paraffin-embedded tissue sections were deparaffinized in xylene (three incubations, 5 min each) and rehydrated through a graded ethanol series (100–10%, 5 min each), followed by three washes in 1× PBS, 5 min each. Antigen retrieval was performed in a decloaking chamber using 1× Antigen Unmasking Solution, Citric Acid Based (Vector Laboratories, Cat #H-3300-250). Endogenous peroxidase activity was quenched with Bloxall (Vector Laboratories, Newark, CA, USA, Cat #SP-6000-100) for 10 min. Sections were then blocked for 40 min using 5% normal goat serum (Vector Laboratories, Cat #S-1000-20). Slides were incubated with the primary anti-HLX antibody (Proteintech, Rosemont, IL, USA) overnight at 4 °C in a humidified chamber. After washing with PBS, sections were incubated for 1 h with a biotinylated secondary antibody (10 µL) combined with 5% normal goat serum in 1× PBS. Following a 5 min wash in 1× PBS, Vectastain ABC reagent (PK-4000; premixed; 25 µL of reagent A and 25 µL of reagent B in 950 µL PBS) was applied for 20 min. Slides were then washed with 1× PBS twice (5 min each), developed using peroxidase substrate (Vector Laboratories, Newark, CA, USA, Cat #SK-4105), and rinsed in water. Hematoxylin counterstaining (Vector Laboratories, Newark, CA, USA, Cat #H-3404-100) was performed for 30 s at RT. After a final water wash, slides were mounted with Vectamount (Vector Laboratories, Newark, CA, USA, Cat #H-5000). Negative controls were processed in parallel using normal rabbit IgG (Vector Laboratories, Cat #I-1000-5) in place of the primary antibody. The images were acquired using ApoTome.2 microscope ZEISS (Carl Zeiss AG, Jena, Germany). HLX immunostaining was quantified in a blinded manner, with the investigator performing the ImageJ analysis unaware of the experimental group assignments during scoring.

### 2.11. Statistical Analysis

All statistical analyses were performed using GraphPad Prism version 10.5.0 (GraphPad Software, LLC). An unpaired *t*-test with Welch’s correction was used for comparisons between two groups. Differences were considered statistically significant at *p* < 0.05. For analyses involving multiple testing, FDR-adjusted *p*-values or q-values < 0.05 were considered statistically significant. Data are presented as mean ± SD unless otherwise indicated.

## 3. Results

### 3.1. HLX Expression Is Elevated During PCa Progression

**Figure 1:**
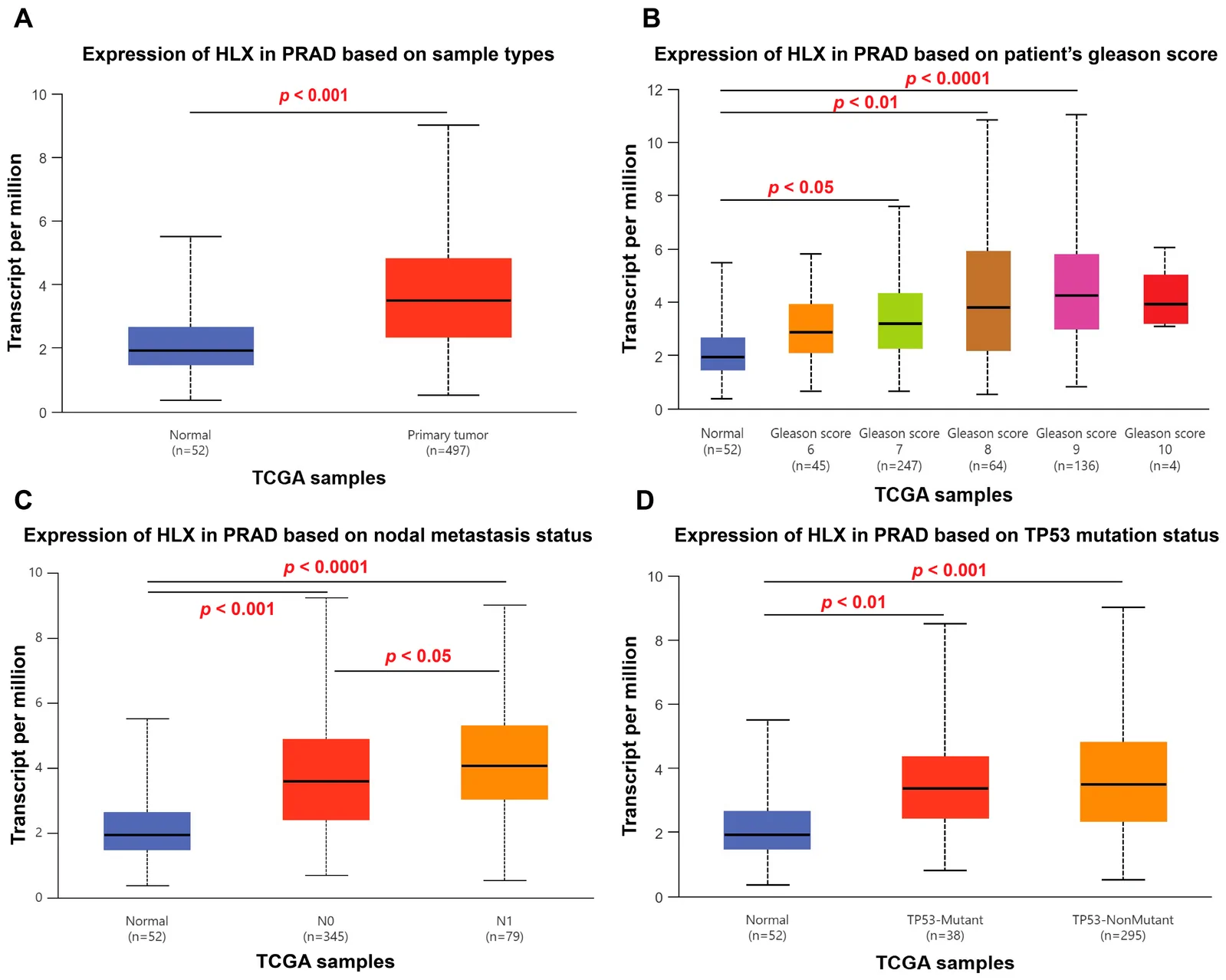
HLX expression is elevated during PCa progression. (**A**) HLX expression was significantly elevated in primary prostate adenocarcinoma (PRAD) relative to normal prostate tissue in the TCGA PanCancer Atlas cohort (*p* < 0.001). (**B**) HLX expression progressively increased with Gleason grade (6–10) and was significantly higher in high-grade tumors compared with low-grade tumors and normal prostate tissue. (**C**) HLX expression was significantly elevated in both node-negative (N0) and node-positive (N1) PRAD tumors relative to normal prostate tissue and was highest in tumors with regional lymph node involvement. Sample distribution: normal (*n* = 52), N0 (*n* = 345), and N1 (*n* = 79). (**D**) HLX expression was significantly elevated in both TP53-mutant and TP53-nonmutant tumors compared with normal prostate tissue. Data was generated using the online tool UALCAN (http://ualcan.path.uab.edu, accessed on 1 November 2025). Statistical significance values for all comparisons are provided in Supplementary Figure S2. Abbreviations: PRAD, prostate adenocarcinoma; TCGA, The Cancer Genome Atlas.

To characterize HLX expression across human malignancies, we first performed a pan-cancer analysis in the TCGA Pan-Cancer Atlas dataset using the UALCAN platform ([36,37]). HLX mRNA expression varied widely across tumor types, with several epithelial cancers exhibiting increased expression relative to corresponding normal tissues (Figure S1A). Focusing on PCa, analysis of the TCGA dataset revealed a significant (*p* < 0.001) upregulation of HLX in primary PRAD (*n* = 497) compared with normal prostate tissues (*n* = 52; Figure 1A). To assess the clinical relevance of this finding, we evaluated the prognostic significance of HLX expression using GEPIA-based Kaplan–Meier survival analysis of TCGA PRAD patients. Notably, high HLX expression (*n* = 246) was associated with a significant (*p* < 0.01) reduction in disease-free survival (Figure S1C). Together, these findings demonstrate that HLX is significantly upregulated in human PCa within the TCGA cohort and suggest that elevated HLX expression may serve as a candidate transcriptomic biomarker associated with aggressive disease phenotypes.

Given the association between HLX expression and reduced disease-free survival, we next investigated whether HLX expression correlates with established indicators of PCa aggressiveness and progression. Using the TCGA PRAD cohort, we first examined HLX expression across tumors stratified by Gleason score (Figure 1B). HLX expression was significantly higher in tumor tissues than in normal prostate tissue and increased progressively with rising Gleason scores (Normal vs. Gleason 7: *p* <0.05; Normal vs. Gleason 8: *p* < 0.01; Normal vs. Gleason 9: *p* < 0.0001). Importantly, high-grade tumors exhibited significantly greater HLX expression than low-grade tumors, as evidenced by significant differences between Gleason 6 vs. Gleason 8 (*p* = 0.0025) and Gleason 6 vs. Gleason 9 (*p* < 0.0001). In addition, the comparison between Gleason 7 and Gleason 8 (*p* = 0.028), and Gleason 7 and Gleason 9 (*p* < 0.0001) tumors were also significant, further supporting a positive association between *HLX* expression and tumor aggressiveness (Figure 1B).

We next assessed *HLX* expression across molecularly defined PRAD subtypes characterized by recurrent genomic alterations, including ETS family fusions (ERG, ETV1/4, FLI1) and mutation-driven subtypes (FOXA1, IDH1, and SPOP; Figure S1B). HLX expression was significantly elevated in FOXA1- and SPOP-mutant tumors compared with normal prostate tissues (Normal vs. FOXA1 mutation, *p* < 0.0001; Normal vs. SPOP mutation, *p* < 0.0001; Figure S1B). Although the direct relationship between these genomic alterations and HLX expression remains unclear, these findings suggest that HLX expression is associated with molecular subtypes linked to aggressive PCa phenotypes. (Figure S1B).

To further examine whether HLX expression correlates with metastatic progression, we analyzed HLX levels in PRAD samples stratified by regional lymph node status (Figure 1C). Although both node-negative (N0) and node-positive (N1) tumors exhibited significantly higher HLX expression than normal prostate tissue (Normal vs. N0, *p* < 0.001; Normal vs. N1, *p* < 0.0001), HLX expression was significantly elevated in N1 tumors relative to N0 tumors (*p* < 0.05), supporting a link between HLX expression and metastatic disease (Figure 1C). We also investigated the relationship between HLX expression and TP53 mutation status in PCa. Both TP53-mutant and TP53-nonmutant tumors exhibited significantly elevated HLX expression compared with normal prostate tissues (TP53-mutant, *p* < 0.01; TP53-nonmutant, *p* < 0.001; Figure 1D). TP53 mutations are frequently associated with genomic instability, tumor progression, and therapeutic resistance in advanced PCa [48]. However, although HLX expression appeared higher in TP53-mutant tumors, no statistically significant difference was observed between TP53-mutant and TP53-nonmutant groups. Taken together, these findings demonstrate that HLX expression increases during PCa progression and is associated with clinically aggressive disease features including higher Gleason grade, metastatic involvement, adverse molecular subtypes, and reduced disease-free survival.

### 3.2. HLX Expression Positively Correlates with TBX2 in Human PCa Cohorts and Cell Lines

**Figure 2:**
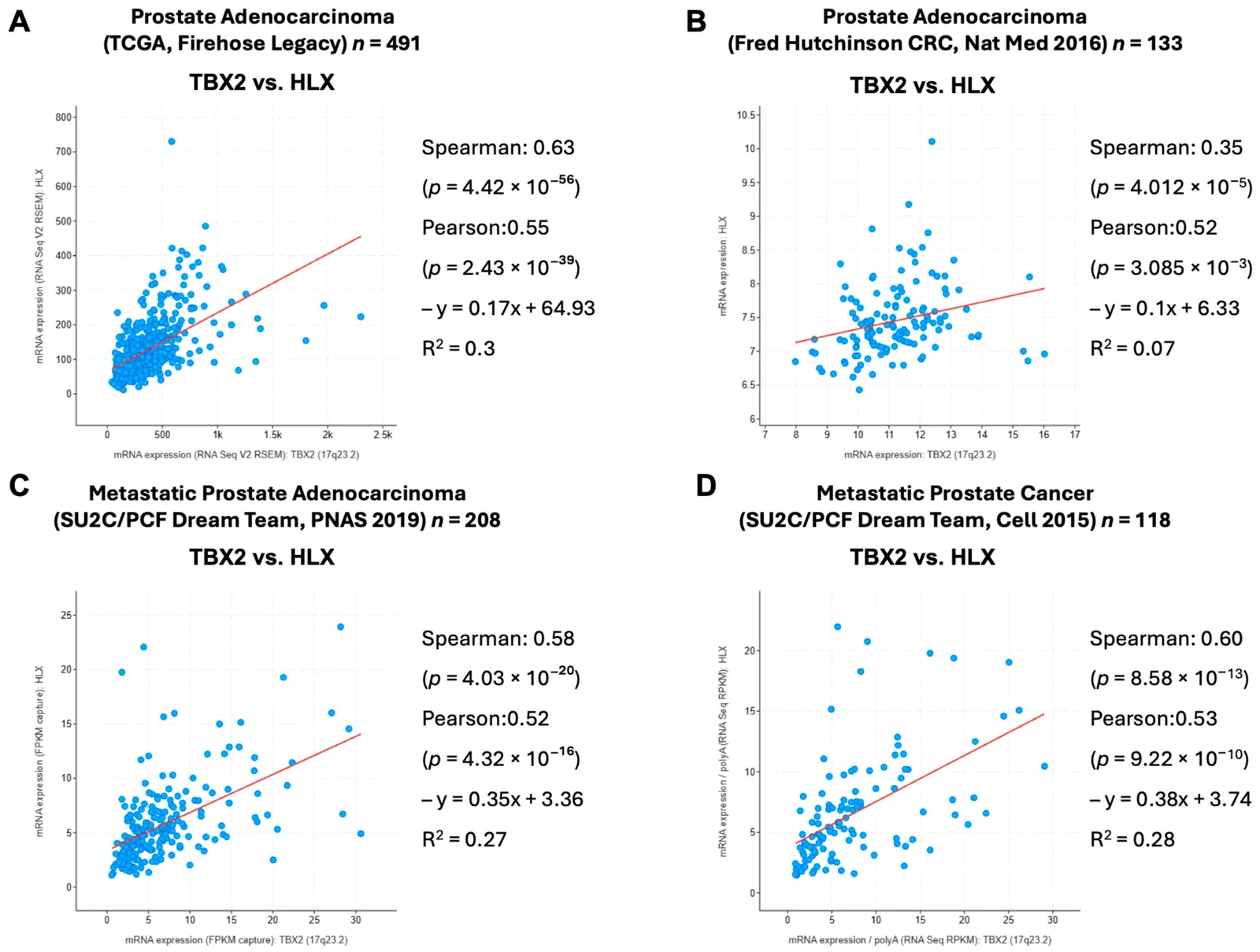
HLX expression positively correlates with TBX2 across human PCa cohorts. (**A**–**D**) HLX mRNA expression positively correlated with TBX2 mRNA expression across human PCa clinical datasets analyzed through cBioPortal, including PRAD (TCGA Firehose Legacy; *n* = 491) [41], PRAD (Fred Hutchinson CRC, *Nature Medicine* 2016; *n* = 133) [42], Metastatic PRAD (SU2C/PCF Dream Team, PNAS 2019; *n* = 208) [43], and Metastatic PCa (SU2C/PCF Dream Team, *Cell* 2015; *n* = 118) [44]. Scatter plots depict Spearman correlations between HLX and TBX2 mRNA expression in each cohort (Spearman r = 0.35–0.63; *p* < 0.001 for all datasets).

Given the association of HLX with aggressive PCa phenotypes, we next sought to identify signaling networks that may contribute to HLX-associated disease progression. Recent studies in group 3 medulloblastoma demonstrated that HLX directly regulates several oncogenic targets, including TBX2 [24]. Importantly, previous studies from our laboratory established TBX2 as a key driver of metastatic progression, lineage plasticity, and therapeutic resistance [27,28,29]. These observations prompted us to investigate whether HLX and TBX2 are functionally linked in human PCa. To address this, we used cBioPortal to perform pairwise correlation analyses of TBX2 and HLX mRNA expression across publicly available PCa datasets encompassing both primary and metastatic disease, including TCGA PRAD, Fred Hutchinson, and SU2C/PCF cohorts [41,42,43,44]. Across all datasets examined, TBX2 mRNA expression showed consistent positive correlations with HLX mRNA expression (Spearman r = 0.35–0.63, *p* < 0.001 for all datasets) (Figure 2A–D) including, TCGA Firehose Legacy (*n* = 491; Spearman r = 0.63, *p* = 4.42 × 10^−56^; Figure 2A), Fred Hutchinson Cancer Research Center, Nat Med 2016 (*n* = 133; Spearman r = 0.35, *p* = 4.01 × 10^−5^; Figure 2B), the expanded SU2C/PCF Dream Team PNAS 2019 (*n* = 208; Spearman r = 0.58, *p* = 4.03 × 10^−20^; Figure 2C), and the original SU2C/PCF Dream Team Cell 2015 (*n* = 118; Spearman r = 0.60, *p* = 8.58 × 10^−13^; Figure 2D).

To determine whether the clinical association between HLX and TBX2 is recapitulated in experimental models, we examined HLX and TBX2 expression across a panel of human PCa cell lines at both the mRNA and protein levels. Elevated HLX mRNA expression was observed in enzalutamide-resistant PCa cell lines (LNCaP^EnzaR^ and C4-2B^EnzaR^) relative to their corresponding parental controls (LNCaP and C4-2B), while other CRPC cell lines including 22Rv1 and PC3 also exhibited increased HLX expression compared to LNCaP, a castrate-sensitive prostate cancer (CSPC) cell line (Figure S2A). Notably, TBX2 mRNA expression was significantly higher in LNCaP^EnzaR^ and PC3 cells relative to the other PCa cell lines examined (Figure S2B). Consistent with the mRNA levels, Western blot analysis demonstrated that HLX and TBX2 protein expression closely paralleled their mRNA levels across the analyzed cell lines. Figure S2C,D). Together, these findings demonstrate that HLX and TBX2 are co-expressed in clinically relevant CSPC and CRPC models, supporting the existence of a shared regulatory network in advanced disease.

### 3.3. HLX- and TBX2-Associated Gene Programs Converge on Pro-Metastatic Pathways in Metastatic PCa

**Figure 3:**
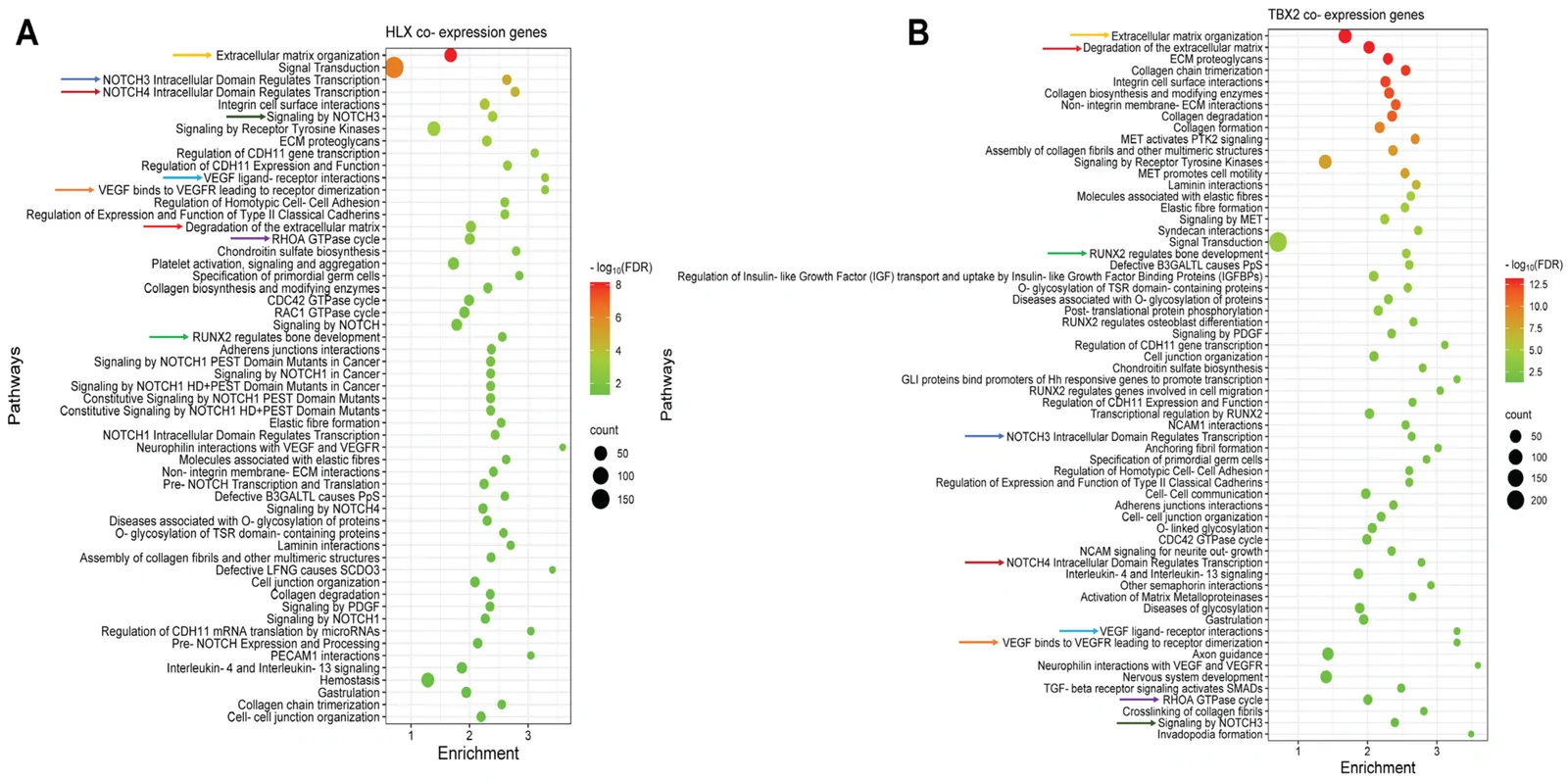
HLX– and TBX2–associated gene programs converge on pro-metastatic pathways in metastatic PCa. (**A**) Reactome pathway enrichment analysis of genes positively correlated with HLX expression in metastatic PRAD from the SU2C/PCF Dream Team cohort (Spearman correlation coefficient r ≥ 0.4, FDR-adjusted q < 0.05, [43]. (**B**) Reactome pathway enrichment analysis of genes positively correlated with TBX2 expression in the same cohort (Spearman correlation coefficient r ≥ 0.4, FDR-adjusted q < 0.05). Dot size represents the number of genes in each pathway, and color intensity reflects statistical significance.

Having established a strong association between HLX and TBX2 expression in human PCa, we next investigated whether these factors are linked to common biological programs in advanced disease. To address this question, we analyzed genes positively correlated with HLX or TBX2 expression in metastatic CRPC samples from the SU2C/PCF Dream Team cohort [43]. Using cBioPortal-based correlation analysis (Spearman correlation coefficient r ≥ 0.4, FDR-adjusted q < 0.05), we identified distinct yet partially overlapping sets of HLX- and TBX2-coexpressed genes. Reactome pathway enrichment showed that both HLX- and TBX2-correlated gene programs were significantly enriched for extracellular matrix organization, cell–cell and cell–matrix adhesion, developmental signaling cascades, and NOTCH signaling (Figure 3A,B). Additional shared pathways included VEGF-mediated MAPK signaling, cytoskeletal remodeling, collagen biosynthesis and degradation, and biological processes linked to lineage plasticity and tissue morphogenesis. Venn diagram analysis confirmed substantial overlap between pathways enriched in HLX- and TBX2-associated gene programs (Figure S3A,B), highlighting convergence on common biological networks. These pathways, including extracellular matrix organization, cell adhesion, developmental signaling, NOTCH signaling, and VEGF-MAPK signaling, are well-established mediators of metastatic progression, tumor–microenvironment interactions, and cellular plasticity in advanced PCa [49,50,51]. Collectively, these findings suggest that HLX and TBX2 expressions are associated with shared transcriptional programs involved in extracellular matrix remodeling, developmental signaling, and cellular plasticity in metastatic PCa. These analyses identify biological programs correlated with HLX and TBX2 expression and do not imply direct transcriptional regulation of the enriched pathways.

### 3.4. HLX Is a Direct Transcriptional Target of TBX2 in Human CRPC Cells

**Figure 4:**
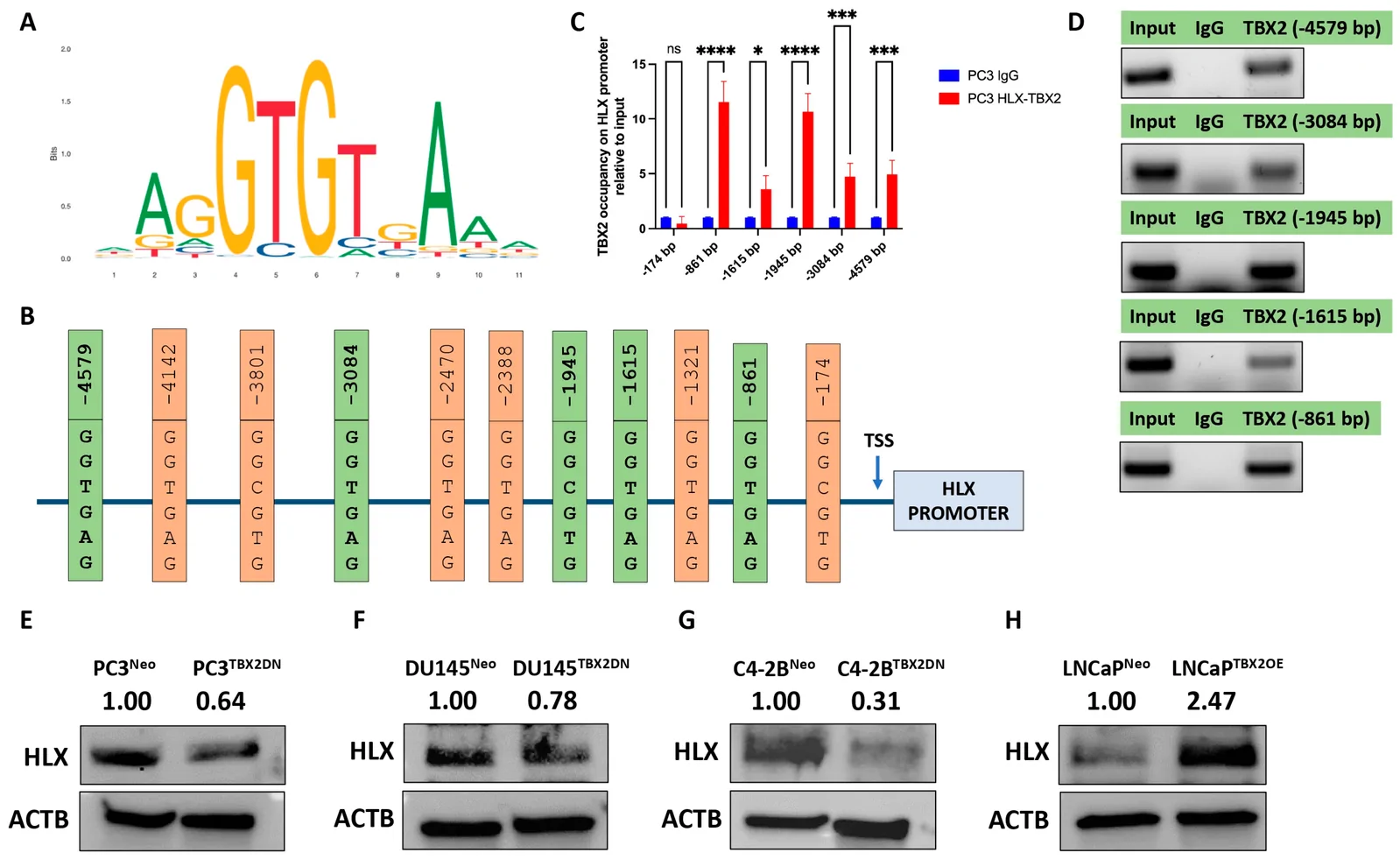
HLX is a direct transcriptional target of TBX2 in human CRPC cells. (**A**) JASPAR-derived consensus T-box binding sequence used for identification of putative TBX2-binding sites within the HLX promoter. (**B**) In silico analysis of the HLX promoter identified multiple putative TBX2-binding sites. (**C**) Quantification of the ChIP assay by qPCR demonstrated 12-fold, 3-fold, 10-fold, 4-fold, and 4.5-fold enrichment at the TBX2-binding sites located at −861 bp, −1615 bp, −1945 bp, −3084 bp, and −4579 bp of the HLX promoter, respectively, normalized to the input control. In contrast, the −174 bp binding site (used as a negative control) did not show any significant enrichment. Two-way ANOVA was performed (*n* = 3); Statistical significance was determined using “Šídák’s multiple comparisons test; * *p* < 0.05, *** *p* < 0.001, and **** *p* < 0.0001. (**D**) Chromatin immunoprecipitation (ChIP) analysis demonstrated TBX2 occupancy at –861 bp, –1615 bp, –1945 bp, –3084 bp, –4579 bp, upstream of the HLX transcription start site (TSS). (**E**–**H**) Western blot analysis of HLX expression in genetically modified PC3, DU145, C4-2B, and LNCaP human PCa cells following TBX2 dominant-negative (DN) or overexpression (OE) modulation. Band intensities were quantified using ImageJ software, normalized to the corresponding β-actin (ACTB) loading controls, and expressed relative to the control group (set to 1). Data are presented as mean ± SD from three independent biological replicates (*n* = 3). Statistical significance was determined using an unpaired two-tailed Student’s *t*-test with Welch’s correction (*p* < 0.05). Abbreviation: TSS, transcription start site.

Because TBX2 and HLX expression were strongly correlated across multiple patient cohorts and PCa cell line models, we next investigated whether HLX is directly regulated by TBX2. We first performed ChIP analyses to identify potential TBX2-binding sites within the HLX promoter. Based on previously reported consensus T-Box binding sequences (Figure 4A; [52]), in silico analysis identified multiple putative TBX2-binding regions within the HLX promoter (Figure 4B). ChIP-PCR and quantification assays confirmed TBX2 occupancy at −4579 bp, −3084 bp, −1945 bp, and −861 bp upstream of the HLX transcription start site (TSS; Figure 4C,D).

To further validate this regulatory relationship, we analyzed the expression of HLX in human PCa cell line models genetically modulated for TBX2 using two complementary approaches. First, TBX2 activity was disrupted by transfecting a dominant-negative (DN) TBX2 construct (TBX2DN) into PC3, DU145, and C4-2B cells, which exhibit relatively high endogenous HLX expression. Second, TBX2 was overexpressed in LNCaP cells, which display relatively low endogenous TBX2 and HLX expressions. In PC3^TBX2DN^, DU145^TBX2DN^, and C4-2B^TBX2DN^ cells, HLX protein levels were significantly reduced compared with their respective control cell lines (PC3^Neo^, DU145^Neo^, and C4-2B^Neo^; Figure 4E–G). Conversely, HLX expression was significantly increased in LNCaP^TBX2OE^ cells relative to LNCaP^Neo^ controls (Figure 4H). Together, these findings support HLX as a direct transcriptional target of TBX2 in PCa cells. These results provide a mechanistic basis for the strong association between TBX2 and HLX observed across human PCa cohorts and experimental models.

### 3.5. HLX Is Associated with Epithelial–Mesenchymal Transition, Extracellular Matrix, and NOTCH-Linked Transcriptional Programs in Advanced PCa

**Figure 5:**
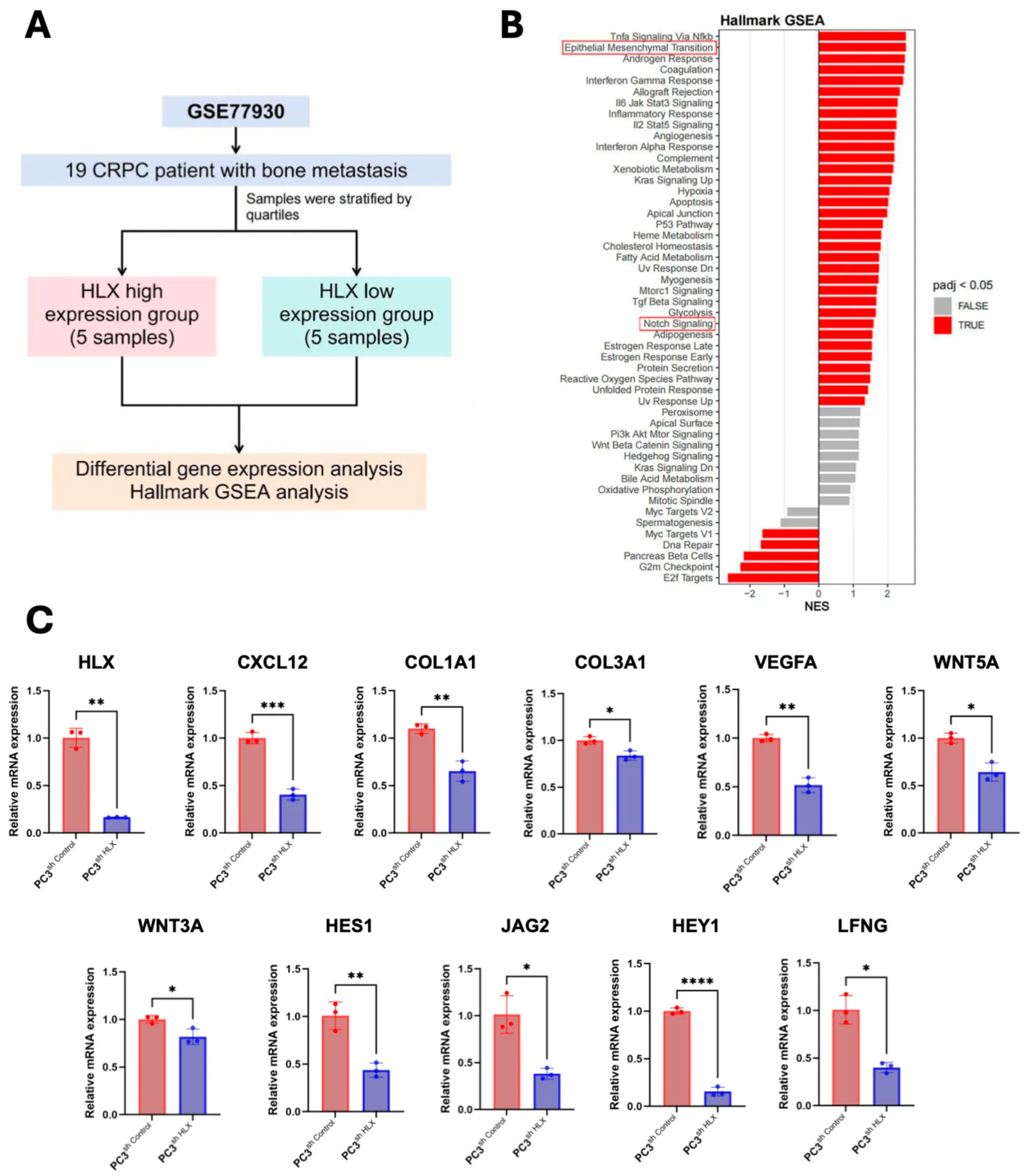
HLX is associated with epithelial–mesenchymal transition, extracellular matrix, and NOTCH-linked transcriptional programs in advanced PCa. (**A**) Schematic workflow illustrating transcriptomic analyses of CRPC bone metastasis samples from the GSE77930 cohort. Nineteen CRPC bone metastasis samples were stratified according to HLX expression using quartile-based grouping. Samples within the upper quartile were designated as the HLX-high group (*n* = 5), whereas samples within the lower quartile were designated as the HLX-low group (*n* = 5). Differential gene expression analysis followed by Hallmark Gene Set Enrichment Analysis (GSEA) was performed between the two groups. (**B**) Hallmark GSEA revealed significant enrichment of epithelial–mesenchymal transition (EMT), NOTCH signaling, TGF-β signaling, IL6-JAK-STAT3 signaling, inflammatory response, angiogenesis, hypoxia, and KRAS signaling pathways in HLX-high CRPC bone metastases. Positive normalized enrichment scores (NES) indicate pathways enriched in the HLX-high group, whereas negative NES values indicate pathways enriched in the HLX-low group. Red bars represent significantly enriched pathways (adjusted *p*-value < 0.05), whereas gray bars indicate non-significant pathways. (**C**) qPCR analyses demonstrating reduced expression of extracellular matrix-associated genes, including CXCL12, COL1A1, COL3A1, VEGFA, and WNT5A, following stable HLX knockdown in PC3 cells compared with shControl cells. qPCR analyses demonstrating reduced expression of NOTCH signaling components and downstream targets, including HES1, JAG2, HEY1, and LFNG, following HLX knockdown in PC3 cells. Relative mRNA expression levels were normalized to β-actin and calculated using the 2^−ΔCt^ method. Data are presented as mean ± SD from three independent biological replicates (*n* = 3). Statistical significance was determined using an unpaired two-tailed Student’s *t*-test with Welch’s correction (* *p* < 0.05, ** *p* < 0.01, *** *p* < 0.001, **** *p* < 0.0001).

To investigate the biological programs associated with elevated HLX expression in advanced PCa, we analyzed transcriptomic profiles from CRPC bone metastases (GSE77930). Samples were stratified into HLX-high and HLX-low groups based on upper and lower quartiles of HLX expression (*n* = 5/group), followed by differential gene expression and Hallmark Gene Set Enrichment Analyses (GSEA) (Figure 5A). Hallmark GSEA demonstrated significant enrichment of multiple pathways implicated in aggressive PCa phenotypes within HLX-high tumors, including epithelial–mesenchymal transition (EMT), NOTCH signaling, and TGF-β signaling (Figure 5B). Additional enriched pathways included androgen response, IL6-JAK-STAT3 signaling, inflammatory response, angiogenesis, hypoxia, and KRAS signaling, suggesting that elevated HLX expression is associated with transcriptional programs linked to cellular plasticity and metastatic disease.

Given the enrichment of EMT- and NOTCH-associated pathways in HLX-high CRPC bone metastases, we next investigated whether HLX contributes to the regulation of genes associated with extracellular matrix remodeling and NOTCH signaling in human PCa cells. Stable shRNA-mediated knockdown of HLX in PC3 cells resulted in significant reductions in the expression of extracellular matrix-associated genes, including CXCL12, COL1A1, COL3A1, VEGFA, and WNT5A, compared with shControl cells (Figure 5C). Similarly, HLX knockdown significantly suppressed the expression of key NOTCH signaling components and downstream targets, including HES1, JAG2, HEY1, and LFNG (Figure 5C).

Collectively, these findings suggest that HLX is associated with transcriptional programs involved in extracellular matrix organization, cellular plasticity, and NOTCH signaling in advanced PCa. The concordance between transcriptomic analyses of CRPC bone metastases and HLX knockdown studies further supports a potential role for HLX in regulating biological pathways linked to aggressive PCa phenotypes.

### 3.6. Reduced HLX Expression Is Associated with Suppression of Metastasis in TBX2DN Orthotopic Xenografts

**Figure 6:**
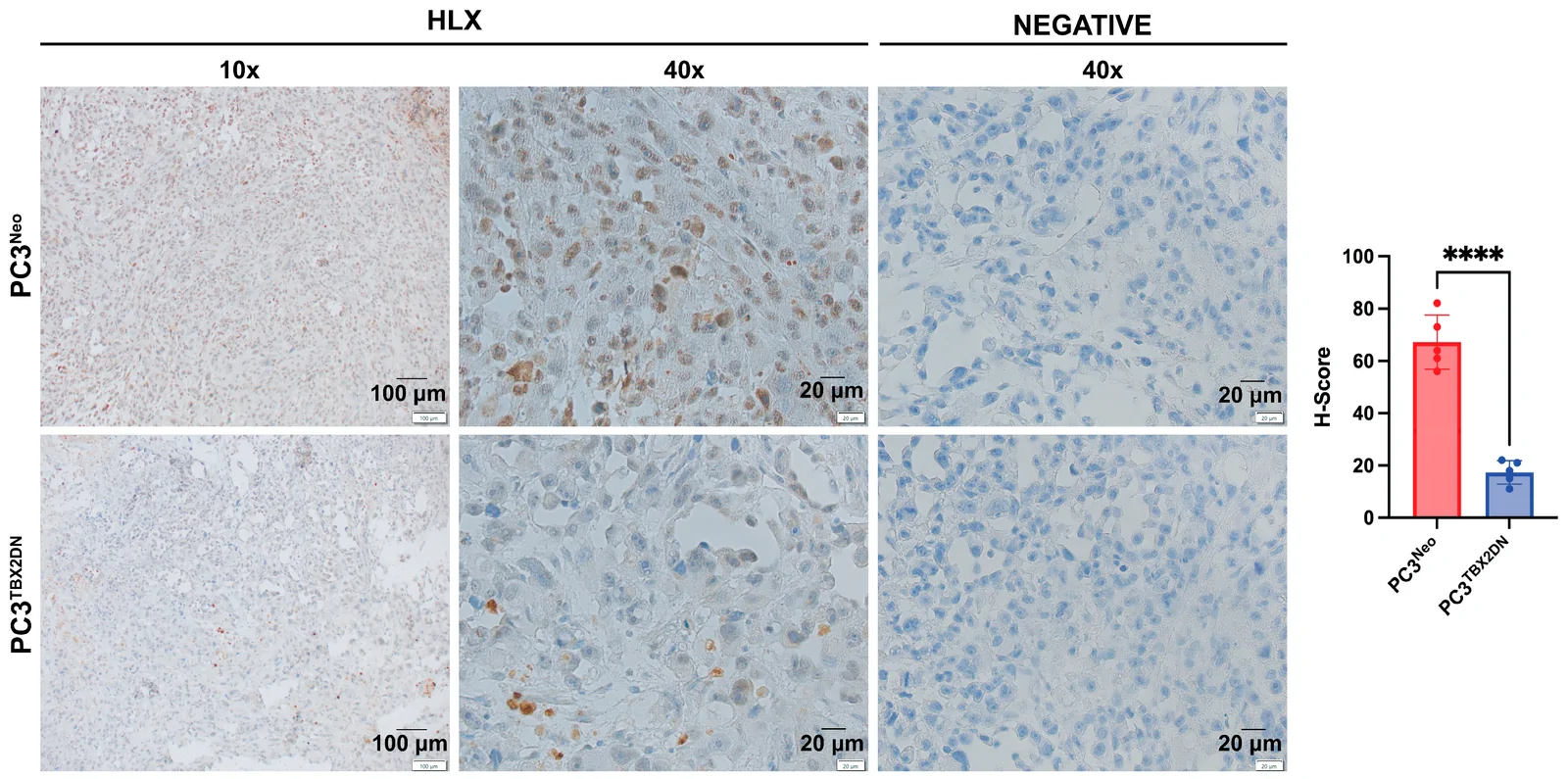
Reduced HLX expression accompanies suppression of metastasis in TBX2DN orthotopic xenografts. Representative immunohistochemical (IHC) staining for HLX in orthotopic xenograft tumors generated from PC3^Neo^ and PC3^TBX2DN^ human PCa cells (*n* = 3 per group). HLX expression was reduced in PC3^TBX2DN^ tumors compared with PC3^Neo^ controls, consistent with TBX2-dependent regulation of HLX and the previously reported suppression of metastatic progression in TBX2DN xenografts. Scale bar for 10× is 100 µm and 40× is 20 µm. Statistical significance was determined using an unpaired two-tailed Student’s *t*-test with Welch’s correction (**** *p* < 0.0001).

Finally, we examined the relationship between TBX2 and HLX in the context of metastatic progression using an orthotopic xenograft mouse model. In a previous study, we demonstrated that dominant-negative disruption of TBX2 activity in PC3 human CRPC cells significantly reduced invasive capacity and abrogated lymph node metastasis *in vivo* [27]. To determine whether HLX expression was affected in this setting, we performed immunohistochemical (IHC) analysis of orthotopic xenograft tumors. HLX expression was markedly reduced in PC3^TBX2DN^ tumors compared with PC3^Neo^ control tumors (*n* = 3; Figure 6). These findings extend our *in vitro* observations and support the *in vivo* relevance of the TBX2-HLX regulatory axis. Collectively, the reduction in HLX expression in TBX2DN tumors, together with suppression of metastatic progression, supports a role for the TBX2-HLX axis in aggressive CRPC phenotypes.

## 4. Discussion

Transcriptional reprogramming is a fundamental hallmark of PCa progression; however, the transcriptional regulators that coordinate these programs remain incompletely defined and underexplored as biomarkers and/or therapeutic targets [53]. In this study, we identify HLX as a potential biomarker of aggressive PCa and establish it as a transcriptional effector downstream of TBX2. By integrating bioinformatic analyses of large-scale PCa clinical datasets with functional studies in human PCa cell lines and an orthotopic xenograft mouse model, we uncover a TBX2–HLX regulatory axis associated with developmental and metastasis-related transcriptional programs in PCa. Across multiple independent clinical cohorts, HLX expression was consistently elevated in PRAD relative to normal prostate tissue and progressively increased with Gleason grade, nodal involvement, and metastatic disease, supporting a role for HLX in disease progression. Consistent with these clinicopathologic associations, elevated HLX was also linked to reduced disease-free survival, further highlighting its potential as a prognostic biomarker in PCa. HLX expression was elevated in both TP53-mutant and TP53-nonmutant tumors relative to normal prostate tissue, suggesting that HLX expression is associated with prostate tumorigenesis irrespective of TP53 mutation status. Collectively, these findings position HLX as a downstream transcriptional effector of TBX2 that is associated with developmental and pro-metastatic transcriptional programs in PCa.

HLX has been reported to function as an oncogenic regulator and prognostic marker in AML and group 3 medulloblastoma, and its expression increases with advancing disease stage in colorectal cancer [15,21,24]. Our data extends these observations to PCa, where elevated HLX expression is associated with nodal metastasis and reduced disease-free survival, supporting its association with aggressive disease phenotypes [15]. These findings extend the broader role of homeobox transcription factors in PCa biology. The well-established association between germline HOXB13 mutations and hereditary PCa risk underscores how disruption of developmental transcriptional programs is a recurring feature of aggressive PCa [22,23].

Although HLX and TBX2 have independently been implicated in oncogenic processes across multiple malignancies, a central mechanistic finding of this study is the identification of HLX as a direct transcriptional target of TBX2 in PCa. Accumulating evidence from our laboratory identifies TBX2 as a central regulator of metastasis, lineage plasticity, and therapy resistance in PCa [27,28,29]; however, the precise downstream transcriptional programs through which it orchestrates these phenotypes is not fully understood. Across multiple independent clinical datasets, we observed a strong and reproducible positive correlation between TBX2 and HLX expression, a relationship that was recapitulated in human PCa cell line models. Genetic modulation experiments provided mechanistic evidence that HLX expression is TBX2-dependent: dominant-negative disruption of TBX2 activity reduced HLX expression, whereas TBX2 overexpression increased HLX expression. Furthermore, ChIP analyses demonstrated TBX2 occupancy at multiple regions of the HLX promoter, supporting direct transcriptional regulation of HLX by TBX2. To our knowledge, this study represents the first report identifying HLX as a direct transcriptional target of TBX2 in PCa. Notably, a recent study in group 3 medulloblastoma reported that HLX directly regulates TBX2 [24], suggesting that the regulatory relationship between these two transcription factors may be context dependent and potentially bidirectional in different cancer contexts. Determining whether HLX also regulates TBX2 in PCa will require further investigation.

Consistent with the strong correlation between HLX and TBX2 expression observed in multiple human PCa cohorts [41,42,43,44], pathway analyses of HLX- and TBX2-associated transcriptional programs revealed convergence on pathways involved in extracellular matrix organization, cell–cell and cell–matrix adhesion, developmental and NOTCH signaling pathways. These processes are closely linked to metastatic progression, cellular plasticity, and tumor–microenvironment interactions [54,55,56,57].

**Figure 7:**
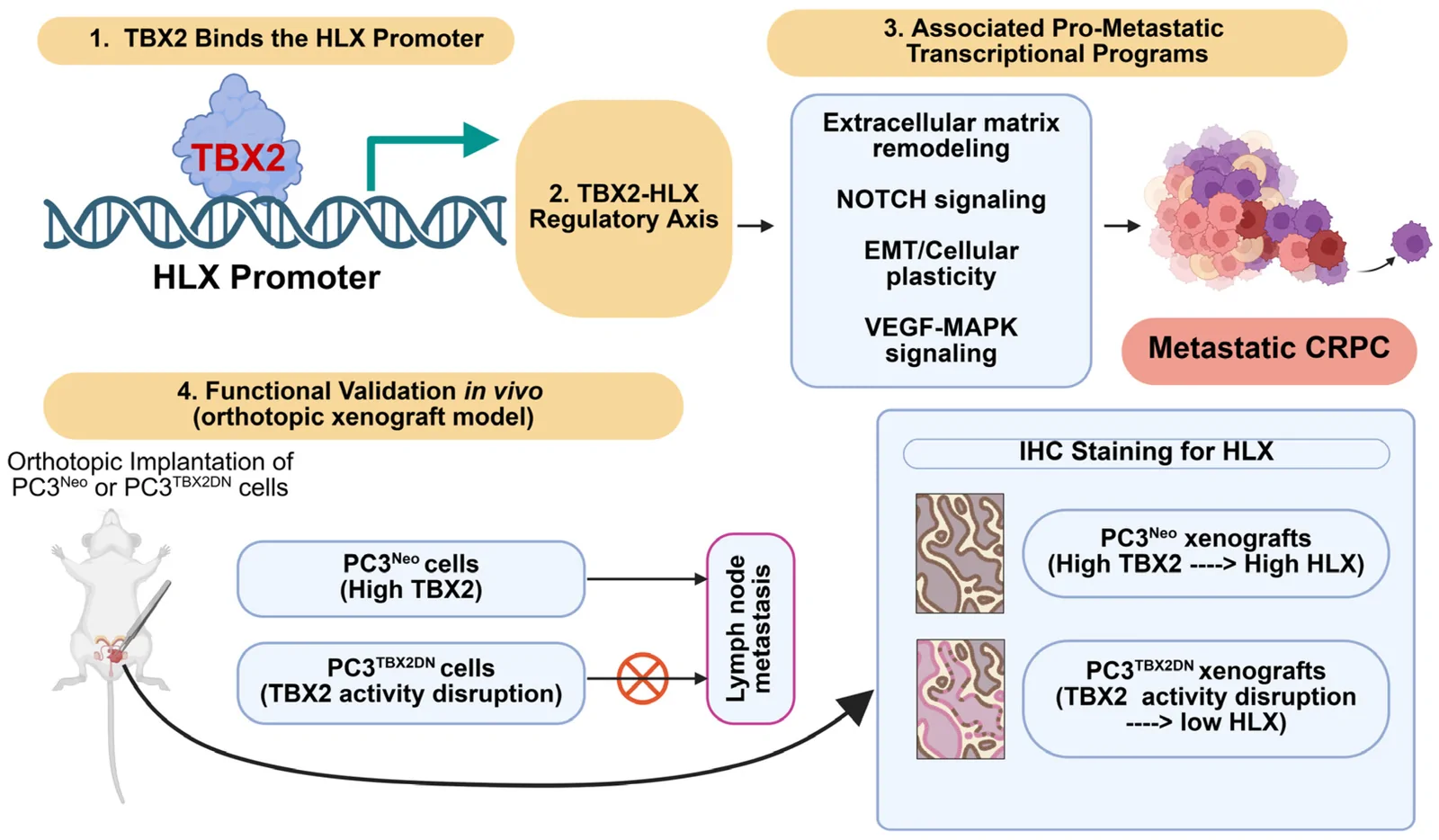
Proposed model of the TBX2–HLX regulatory axis in advanced PCa. TBX2 directly binds the HLX promoter and positively regulates HLX expression in PCa cells. Activation of the TBX2–HLX regulatory axis is associated with pro-metastatic transcriptional programs linked to aggressive disease progression and metastatic CRPC. Functional validation in an orthotopic xenograft model demonstrated that disruption of TBX2 activity using a dominant-negative TBX2 construct (TBX2DN) suppresses HLX expression and reduces metastatic progression, supporting the *in vivo* relevance of the TBX2–HLX axis in advanced PCa. The schematic summarizes the central findings of this study and highlights the TBX2–HLX regulatory axis as a potential driver of metastatic CRPC.

The acquisition of metastatic competence in advanced PCa is accompanied by extensive transcriptional reprogramming involving epithelial–mesenchymal plasticity, extracellular matrix remodeling, and developmental signaling pathways [58]. In the present study, Hallmark GSEAs demonstrated that HLX-high CRPC bone metastases were enriched for EMT-, extracellular matrix-, and NOTCH-associated transcriptional programs. Consistent with these findings, HLX knockdown in PC3 cells resulted in reduced expression of genes implicated in extracellular matrix organization (CXCL12, COL1A1, COL3A1, VEGFA, and WNT5A) and NOTCH signaling (HES1, JAG2, HEY1, and LFNG). These observations suggest HLX contributes to transcriptional programs associated with aggressive PCa phenotypes. Although the current study does not establish whether HLX is necessary or sufficient for metastatic progression, our findings support HLX as a downstream effector of TBX2 associated with biological pathways linked to cellular plasticity in advanced PCa. Additional mechanistic studies will be required to determine the extent to which HLX functionally mediates TBX2-dependent PCa progression. Collectively, these findings identify HLX as a direct transcriptional effector of TBX2 and support the existence of a TBX2-HLX regulatory axis associated with aggressive and metastasis-related transcriptional states in PCa (Figure 7). In support of this model, dominant-negative disruption of TBX2 reduced HLX expression both *in vitro* and in orthotopic xenograft tumors, further demonstrating the *in vivo* relevance of this regulatory relationship. These findings may also have important clinical implications.

From a clinical perspective, HLX may serve as a potential biomarker of aggressive PCa. Given the association of HLX and TBX2 with EMT, extracellular matrix remodeling, NOTCH signaling, and VEGF/MAPK-related pathways, further investigation of these signaling networks may provide insights into therapeutic vulnerabilities in advanced PCa. Future studies should define the direct genomic targets of HLX, elucidate its cooperation with other lineage-determining transcription factors, and determine whether the TBX2–HLX axis contributes to therapeutic resistance in advanced PCa. Additional mechanistic studies, including rescue experiments and protein-level validation in human clinical specimens, will also be important to establish whether HLX functionally mediates TBX2-dependent PCa progression and to further define its downstream genomic targets in CRPC.

## 5. Conclusions

Our findings identify HLX as a direct transcriptional target and downstream effector of TBX2 in advanced PCa and support the existence of a TBX2-HLX regulatory axis associated with aggressive disease progression and metastasis (Figure 7). HLX expression was elevated in clinically aggressive PCa subtypes and associated with reduced disease-free survival. Furthermore, HLX and TBX2 expression demonstrated a strong positive correlation across multiple independent primary and metastatic PCa cohorts, supporting their coordinated involvement in advanced PCa. Mechanistically, ChIP and genetic modulation studies identified that TBX2 directly regulates HLX expression. Together, these findings establish HLX as a candidate biomarker of aggressive PCa and highlight the TBX2–HLX axis as a potential therapeutic target in advanced PCa.

## Data Availability

This study analyzed publicly available datasets, including those available through cBioPortal and other publicly accessible repositories as cited in the manuscript.

## Supplementary Materials

The following supporting information can be downloaded at https://www.mdpi.com/article/10.3390/biomedicines14081865/s1. Supplementary Figure S1: HLX expression is elevated in human PCa and is associated with reduced disease-free survival. Supplementary Table S1: Statistical significance analyses associated with HLX expression across PCa clinical and molecular subgroups. Supplementary Figure S2: TBX2 and HLX are positively co-expressed in human PCa cell lines. Supplementary Table S2: List of primers used in this study. Supplementary Figure S3: HLX- and TBX2-associated gene programs converge on pro-metastatic pathways in metastatic PCa. Supplementary Table S3: HLX associated gene signature list. Supplementary Table S4: TBX2 associated gene signature list.

## Abbreviations

The following abbreviations are used in this manuscript:

ABC	Avidin–Biotin Complex
ACTB	Beta-actin/β-actin
AML	Acute Myeloid Leukemia
ATCC	American Type Culture Collection
bp	Base Pair
cBioPortal	cBio Cancer Genomics Portal
cDNA	Complementary DNA
ChIP	Chromatin Immunoprecipitation
CO_2_	Carbon Dioxide
COL1A1	Collagen Type I Alpha 1 Chain
COL3A1	Collagen Type III Alpha 1 Chain
CRC	Cancer Research Center
CRPC	Castration-Resistant Prostate Cancer
CSPC	Castration-Sensitive Prostate Cancer
CXCL12	C-X-C Motif Chemokine Ligand 12
DMEM	Dulbecco’s Modified Eagle Medium
DN	Dominant Negative
DNA	Deoxyribonucleic Acid
EDTA	Ethylenediaminetetraacetic Acid
EMT	Epithelial–Mesenchymal Transition
ERG	ETS-Related Gene
ETS	E26 Transformation-Specific
ETV1	ETS Variant Transcription Factor 1
ETV4	ETS Variant Transcription Factor 4
FBS	Fetal Bovine Serum
FLI1	Friend Leukemia Integration 1 Transcription Factor
GEPIA	Gene Expression Profiling Interactive Analysis
GFP	Green Fluorescent Protein
HES1	Hes Family BHLH Transcription Factor 1
HEY1	Hairy/enhancer-of-split related with YRPW motif protein 1
HLX	H2.0-Like Homeobox
HOXB13	Homeobox B13
HRP	Horseradish Peroxidase
IACUC	Institutional Animal Care and Use Committee
IDH1	Isocitrate Dehydrogenase 1
IDT	Integrated DNA Technologies
IgG	Immunoglobulin G
IHC	Immunohistochemistry
JAG2	Jagged Canonical Notch Ligand 2
JAK	Janus Kinase
JASPAR	Joint Academic Software Platform for Regulatory Analysis
LFNG	Lunatic Fringe
LIN9	Lin-9 DREAM MuvB Core Complex Component
MAPK	Mitogen-Activated Protein Kinase
MEM	Minimum Essential Medium
MET500	Metastatic Tumor Project 500
mRNA	Messenger Ribonucleic Acid
MYC	MYC Proto-Oncogene
NIH	National Institutes of Health
NKL	NK-Like Homeobox
OE	Overexpression
PBS	Phosphate-Buffered Saline
PCa	Prostate Cancer
PCR	Polymerase Chain Reaction
PNAS	Proceedings of the National Academy of Sciences
PPI	Protease and Phosphatase Inhibitor
PRAD	Prostate Adenocarcinoma
PS	Penicillin–Streptomycin
PVDF	Polyvinylidene Fluoride
qPCR	Quantitative Polymerase Chain Reaction
RIPA	Radioimmunoprecipitation Assay
RNA	Ribonucleic Acid
RNase	Ribonuclease
RPMI	Roswell Park Memorial Institute
RT	Room Temperature
SCBT	Santa Cruz Biotechnology
SD	Standard Deviation
SDS	Sodium Dodecyl Sulfate
SDS-PAGE	Sodium Dodecyl Sulfate–Polyacrylamide Gel Electrophoresis
SPOP	Speckle-Type POZ Protein
STAT	Signal Transducer and Activator of Transcription
SU2C	Stand Up To Cancer
SYBR	Sytox Green-Based DNA Binding Dye
TBS-T	Tris-Buffered Saline with Tween-20
TBX2	T-Box Transcription Factor 2
TCGA	The Cancer Genome Atlas
TE	Tris-EDTA
TH1	Type 1 Helper T Cell
TP53	Tumor Protein p53
TSS	Transcription Start Site
UALCAN	University of Alabama at Birmingham Cancer Data Analysis Portal
UV	Ultraviolet
VEGF	Vascular Endothelial Growth Factor
VEGFA	Vascular Endothelial Growth Factor A
VUMC	Vanderbilt University Medical Center
WNT5A	Wnt Family Member 5A
