# Supplementary material for: A TBX2-HLX Regulatory Axis Is Associated with Advanced Prostate Cancer
Source: Biomedicines. 2026 Aug 20;14(8):1865. doi: 10.3390/biomedicines14081865 (PMC13509987; doi:10.3390/biomedicines14081865)
Supplement: Supplementary file 1 [file biomedicines-14-01865-s001.zip › biomedicines-4421302-supplementary.pdf]

biomedicines

MDPI

Supplementary Information

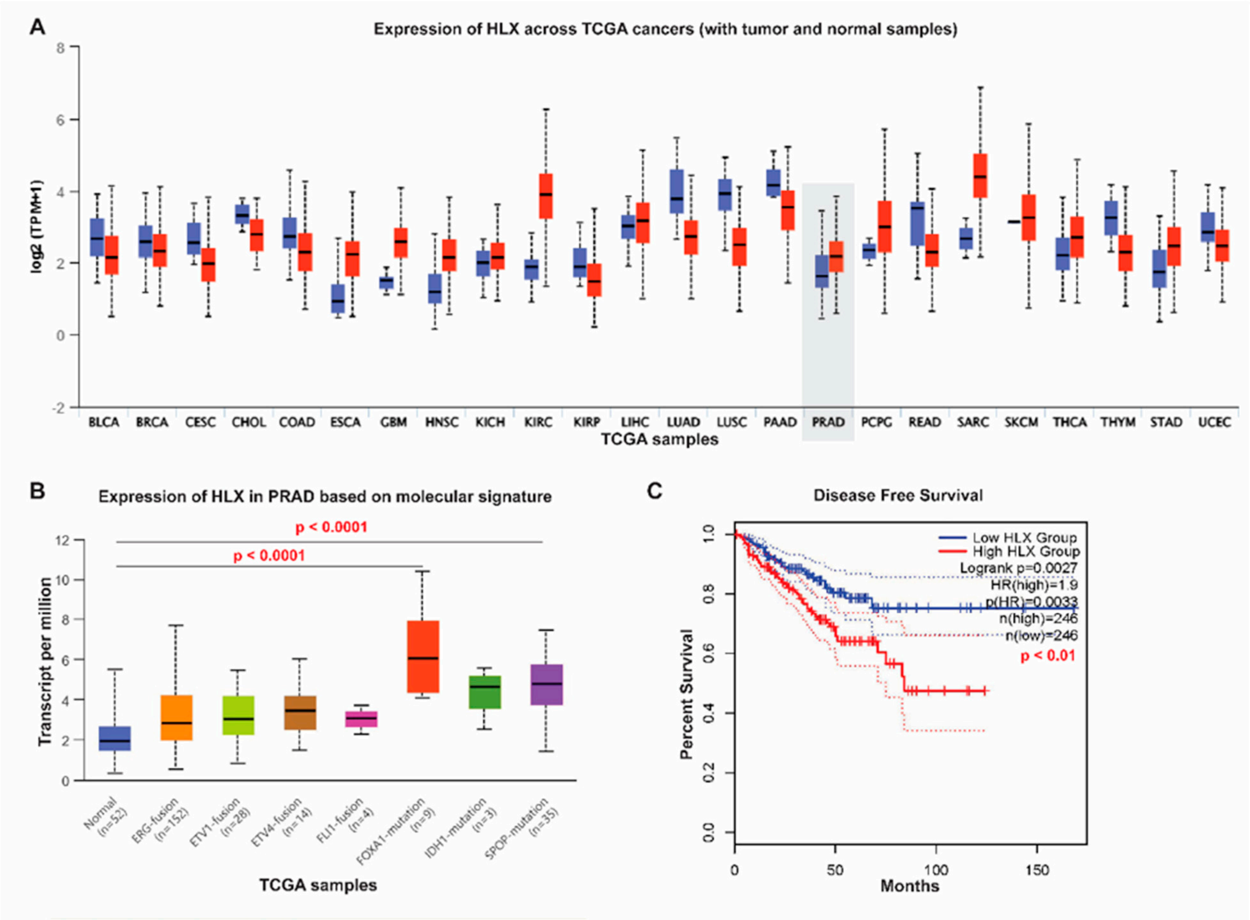

Figure S1. HLX expression is elevated in human PCa and is associated with reduced disease-free survival. (**A**) Pan-cancer analysis of HLX mRNA expression across TCGA tumor and normal tissue datasets. (B**)** HLX expression across TCGA PRAD molecular subtypes defined by recurrent genomic alterations, including ERG, ETV1/4, FLI1, FOXA1, IDH1, and SPOP fusion or mutation status. HLX expression was significantly elevated in fusion- and mutation-positive tumors relative to normal prostate tissue and was further enriched in mutation-driven subtypes compared with ETS fusion–positive tumors. The results were generated from the online tool UALCAN (http://ualcan.path.uab.edu). (**C**) Kaplan–Meier analysis of disease-free survival in TCGA-PRAD patients stratified by HLX expression (n = 246). Patients were divided into HLX-high and HLX-low groups using the median expression cut-off (50%:50%). Survival curves were generated using the GEPIA web server, statistical significance was determined by the log-rank test, and hazard ratios were estimated using the Cox proportional hazards (Cox PH) model [38]. Patients with high HLX expression exhibited significantly shorter disease-free survival than those with low HLX expression (p < 0.01). Statistical significance values for all comparisons are provided in Supplementary Table T1. Abbreviations: PRAD, prostate adenocarcinoma; GEPIA, Gene Expression Profiling Interactive Analysis.

Table S1. Statistical significance analyses associated with HLX expression across PCa clinical and molecular subgroups.

Comparison	Statistical significance
Normal-vs-Primary	4.026500E-04

Comparison	Statistical significance
Normal-vs-Gleason score 6	4.004400E-01
Normal-vs-Gleason score 7	1.091540E-02
Normal-vs-Gleason score 8	1.119850E-03
Normal-vs-Gleason score 9	2.91099999660815E-08
Normal-vs-Gleason score 10	2.203000E-01
Gleason score 6-vs-Gleason score 7	7.877800E-02
Gleason score 6-vs-Gleason score 8	2.497300E-03
Gleason score 6-vs-Gleason score 9	8.33220004103907E-09
Gleason score 6-vs-Gleason score 10	1.587760E-01
Gleason score 7-vs-Gleason score 8	2.849900E-02
Gleason score 7-vs-Gleason score 9	6.13739999932861E-08
Gleason score 7-vs-Gleason score 10	4.946000E-01
Gleason score 8-vs-Gleason score 9	6.830200E-02
Gleason score 8-vs-Gleason score 10	9.192400E-01
Gleason score 9-vs-Gleason score 10	5.400400E-01

Comparison	Statistical significance
Normal-vs-N0	2.565800E-04
Normal-vs-N1	5.73509999999278E-05
N0-vs-N1	4.664500E-02

Comparison	Statistical significance
Normal-vs-TP53-Mutant	3.420200E-03
Normal-vs-TP53-NonMutant	4.799800E-04
TP53-Mutant-vs-TP53-NonMutant	5.737200E-01

Comparison	Statistical significance
Normal-vs-Age(41-60Yrs)	N/A
Normal-vs-Age(61-80Yrs)	N/A
Age(41-60Yrs)-vs-Age(61-80Yrs)	3.971500E-02

Comparison	Statistical significance
Normal-vs-ERG fusion	1.070400E-01
Normal-vs-ETV1 fusion	2.773600E-01
Normal-vs-ETV4 fusion	1.576010E-01
Normal-vs-FLI1 fusion	1.149850E-01
Normal-vs-FOXA1 mutation	3.73000000000179E-05

Normal-vs-IDH1 mutation	2.888600E-01
Normal-vs-SPOP mutation	1.54184999999751E-05
ERG fusion-vs-ETV1 fusion	8.812600E-01
ERG fusion-vs-ETV4 fusion	4.705400E-01
ERG fusion-vs-FLI1 fusion	4.868600E-01
ERG fusion-vs-FOXA1 mutation	3.53600000002618E-06
ERG fusion-vs-IDH1 mutation	4.353400E-01
ERG fusion-vs-SPOP mutation	1.63597999999921E-05
ETV1 fusion-vs-ETV4 fusion	3.847000E-01
ETV1 fusion-vs-FLI1 fusion	4.740400E-01
ETV1 fusion-vs-FOXA1 mutation	3.95970000000023E-05
ETV1 fusion-vs-IDH1 mutation	3.122200E-01
ETV1 fusion-vs-SPOP mutation	3.566100E-04
ETV4 fusion-vs-FLI1 fusion	6.143000E-01
ETV4 fusion-vs-FOXA1 mutation	3.064400E-03
ETV4 fusion-vs-IDH1 mutation	6.334400E-01
ETV4 fusion-vs-SPOP mutation	3.424000E-02
FLI1 fusion-vs-FOXA1 mutation	3.178200E-01
FLI1 fusion-vs-IDH1 mutation	8.221000E-01
FLI1 fusion-vs-SPOP mutation	9.159000E-01
FOXA1 mutation-vs-IDH1 mutation	1.582980E-01
FOXA1 mutation-vs-SPOP mutation	4.538300E-02
IDH1 mutation-vs-SPOP mutation	5.092200E-01

Summary of statistical analyses corresponding to HLX expression comparisons across multiple clinical and molecular categories of PCa. Comparisons include HLX expression across Gleason score groups, molecular subtypes defined by FOXA1 and SPOP mutations and ETS-family fusions, lymph node status (N0 vs. N1), ERG fusion status, and TP53 mutation status in TCGA PRAD datasets. P values for all pairwise comparisons presented in Figures 1 and S1 are provided.

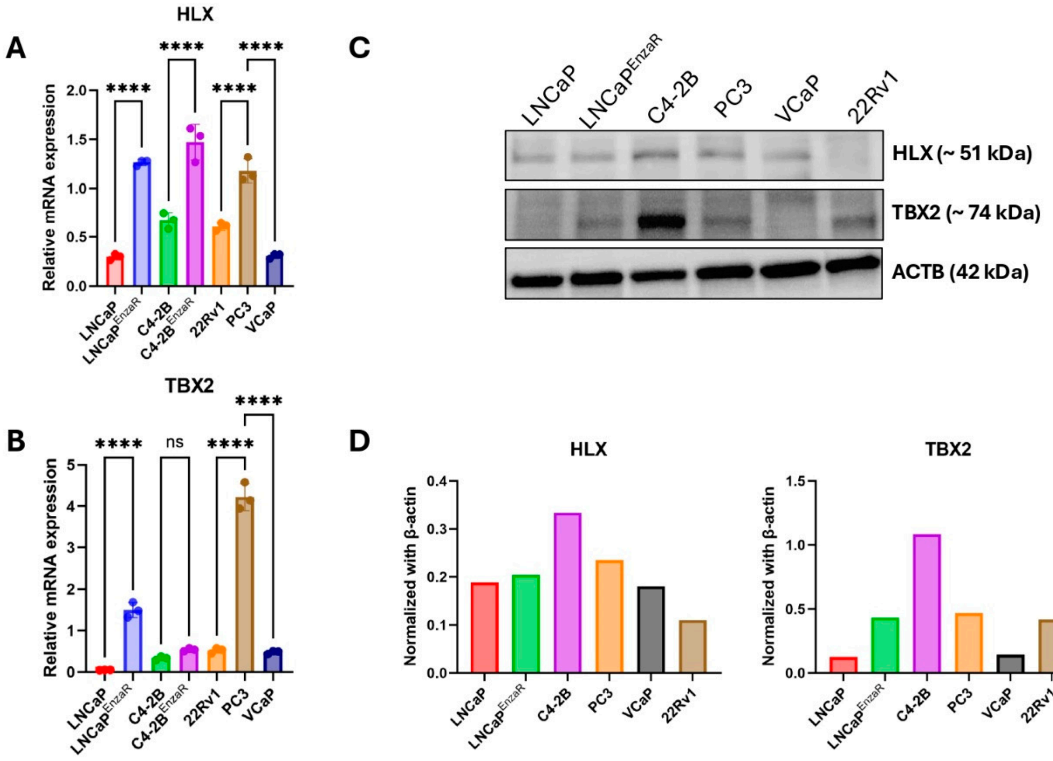

Figure S2. TBX2 and HLX are positively co-expressed in human PCa cell lines. Quantification of HLX (**A**) and TBX2 (**B**) mRNA expression across human PCa cell lines, including LNCaP, LNCaP^{EnzaR}, C4-2B, C4-2B^{EnzaR}, 22Rv1, PC3, and VCaP. HLX and TBX2 mRNA levels were normalized to β-actin (ACTB). (**C**) Western blot analysis of HLX and TBX2 protein expression in LNCaP, LNCaP^{EnzaR}, C4-2B, PC3, VCaP, and 22Rv1 cells. (**D**) Band intensities were quantified using ImageJ, and HLX and TBX2 protein levels were normalized to β-ACTIN. Graphs were generated using GraphPad Prism.

Table S2. List of primers used in this study.

Primer Name	Sequence	Experiment
hHLX F	TCTCACTTCCCTGCTAACCG	
hHLX R	TAGGGACCTGGAAACGTGTC	
hTBX2 F	GGGAGCAGTGGATGGCTAAG	
hTBX2 R	ATGTCGTTGGCTCGCACTAT	
hCXCL12 F	TGCTTACCCGCAAAAGACAA	
hCXCL12 R	GGCTTCAGAGGCAATCACAAA	
hCOL1A1 F	TCTAGACATGTTCAGCTTTGTGGAC	
hCOL1A1 R	CTGTACGCAGGTGATTGGTG	
hCOL3A1 F	CGGCAATCCTGAACTTCCTG	
hCOL3A1 R	ATCAGCTTCAGGGCCTTCTT	
hVEGFA F	GGCCAGCACATAGGAGAGAT	
hVEGFA R	ACGCTCCAGGACTTATACCG	
hWNT5A F	AGCCCAGCTGATTCTTAATACC	
hWNT5A R	GCTCAACTACATGGGACTTTCT	
hWNT3A F	AGGTAAGCTACTCCCTCAACTA	
hWNT3A R	CTGAAGCACCCTCTCATGTATC	
hHES1 F	TCAACACGACACCGGATAAAC	
hHES1 R	GCCGCGAGCTATCTTTCTTCA	
hJAG2 F	TGGGACTGGGACAACGATAC	

hJAG2 R	AGTGGCGCTGTAGTAGTTCTC	qPCR
hHEY1 F	ATCTGCTAAGCTAGAAAAAGCCG	
hHEY1 R	GTGCGCGTCAAAGTAACCT	
hLFNG F	GCCTGCTTGGAGGAAGGATT	
hLFNG R	AGAAAGGCACCTACGCATCG	
hTBX2 HLX ChIP F1 (-4579)	TCACCTACTCCTCCTTCTCCTA	ChIP
hTBX2 HLX ChIP R1(-4579)	AGACCCCAAATCTCATAAAACCT	
hTBX2 HLX ChIP F2(-4142)	TGTGAGAGAGAGAGAGAGGTACA	
hTBX2 HLX ChIP R2(-4142)	GTGGCCAGCTCTCTATTTGG	
hTBX2 HLX ChIP F3(-3801)	CAAAGTATGCGTGTGTGGGT	
hTBX2 HLX ChIP R3(-3801)	ATTGTGCCTGTCTTTCTGCC	
hTBX2 HLX ChIP F4(-3084)	GGTGAAATCGTCTTTGGCTGT	
hTBX2 HLX ChIP R4(-3084)	ACTTGAGCTTGGTCTACGCA	
hTBX2 HLX ChIP F5(-2470)	GCCGGTGGAGATAAAGTTGC	
hTBX2 HLX ChIP R5(-2470)	GAACCAAGAAAGCAGCAGGA	
hTBX2 HLX ChIP F6(-2388)	GGAGTCTGCGGGCTCAG	
hTBX2 HLX ChIP R6(-2388)	CCTCCCTTTCTCCGCCAG	
hTBX2 HLX ChIP F7(-1945)	TCCGGGCCTTGAATCTCG	
hTBX2 HLX ChIP R7(-1945)	CGGTTGTGAAAGAGGTGCAT	
hTBX2 HLX ChIP F8(-1615)	GGCTGAGGTCTAAATGTTTGCA	
hTBX2 HLX ChIP R8(-1615)	GTTTTCTTCCCCTGCTCCA	
hTBX2 HLX ChIP F9(-1321)	AGAGATCTTAGTGCTGGCTGA	
hTBX2 HLX ChIP R9(-1321)	ACTTTTGTTTTCGATTGCCCTAA	
hTBX2 HLX ChIP F10(-861)	CGGAAATCTGCTCTTGTGGG	
hTBX2 HLX ChIP R10(-861)	TCTGAGCTGTCACACAGTGAC	
hTBX2 HLX ChIP F11(-174)	GAAGAGAGTCAGAGCCCTCC	
hTBX2 HLX ChIP R11(-174)	GGGAGTGGAGGGGAATTAGG	

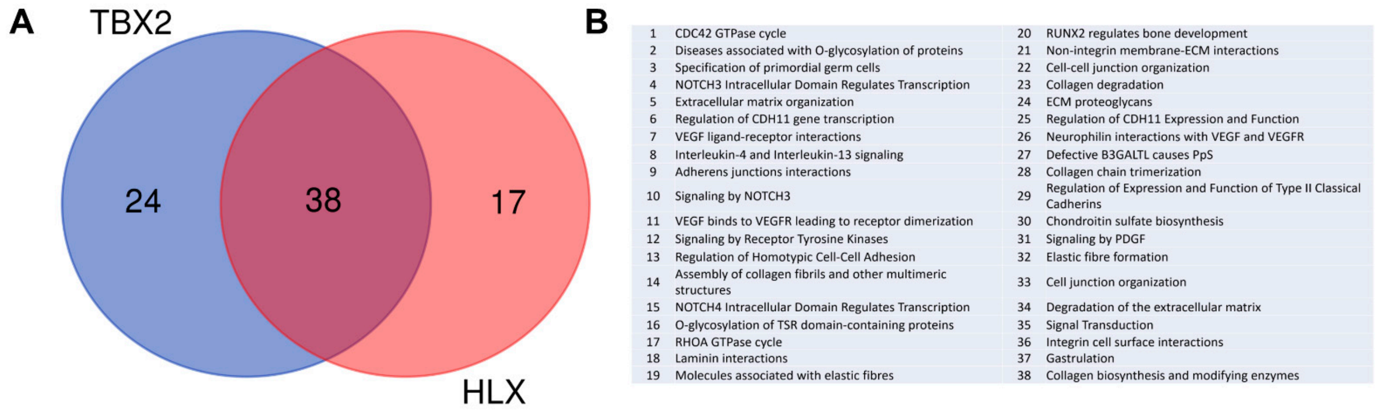

Figure S3. HLX- and TBX2-associated gene programs converge on pro-metastatic pathways in metastatic PCa. (**A**) Venn diagram illustrating the overlap of Reactome pathways enriched in HLX- and TBX2-associated gene signatures. (**B**) Shared pathways identified from the overlap between HLX- and TBX2-associated gene signatures shown in panel (A). The Reactome pathways enriched in the HLX- and TBX2-associated gene signature lists are provided in Supplementary Tables T3 (HLX) and T4 (TBX2).
